# Early Stage Forest Fire Detection from Himawari-8 AHI Images Using a Modified MOD14 Algorithm Combined with Machine Learning

**DOI:** 10.3390/s23010210

**Published:** 2022-12-25

**Authors:** Naoto Maeda, Hideyuki Tonooka

**Affiliations:** Graduate School of Science and Engineering, Ibaraki University, Hitachi 3168511, Japan

**Keywords:** forest fire, fire detection, thermal anomaly, MOD14 algorithm, geostationary satellite, Himawari-8, AHI, machine learning, random forest, contextual classification

## Abstract

The early detection and rapid extinguishing of forest fires are effective in reducing their spread. Based on the MODIS Thermal Anomaly (MOD14) algorithm, we propose an early stage fire detection method from low-spatial-resolution but high-temporal-resolution images, observed by the Advanced Himawari Imager (AHI) onboard the geostationary meteorological satellite Himawari-8. In order to not miss early stage forest fire pixels with low temperature, we omit the potential fire pixel detection from the MOD14 algorithm and parameterize four contextual conditions included in the MOD14 algorithm as features. The proposed method detects fire pixels from forest areas using a random forest classifier taking these contextual parameters, nine AHI band values, solar zenith angle, and five meteorological values as inputs. To evaluate the proposed method, we trained the random forest classifier using an early stage forest fire data set generated by a time-reversal approach with MOD14 products and time-series AHI images in Australia. The results demonstrate that the proposed method with all parameters can detect fire pixels with about 90% precision and recall, and that the contribution of contextual parameters is particularly significant in the random forest classifier. The proposed method is applicable to other geostationary and polar-orbiting satellite sensors, and it is expected to be used as an effective method for forest fire detection.

## 1. Introduction

Monitoring forest fires is important, as they are disasters affecting air pollution, ecosystem changes, and climate change. One way to monitor forest fires is to build a sensor network in the field and analyze the information obtained from these sensors to detect fires. For example, Low-Power Wide Area Network (LPWAN) technology has attracted attention as a fire detection solution, due to its low-speed but long-distance data transmission and long battery life [1,2,3]. On the other hand, satellite remote sensing has also attracted attention as a forest fire detection means, which can cover a wider area and does not require local sensors.

In forest fire detection by remote sensing, a combination of the 4 µm and 11 µm bands is often used to separate the temperature change caused by the fire from the temperature in the background area. The MODIS fire product [4,5] is a typical product that utilizes this technique, employing the MOD14 algorithm as a method for fire detection. This is based on a context test, in which the pixel of interest is compared with surrounding pixels to ensure stable detection, regardless of the characteristics of the region. In this algorithm, a threshold judgment is first made regarding the mean value and standard deviation of the brightness temperature of the surrounding area of the pixel of interest, and pixels with even a slight possibility of fire are determined as potential fires. Then, multiple context tests are performed on these pixels, in order to determine whether they are fires or not. Finally, several threshold judgments are used to eliminate false positives. The MOD14 algorithm has been applied to satellite sensors other than MODIS, and it has been shown to provide high accuracy [6,7,8]. In addition, context-based fire detection algorithms such as this algorithm are traditional methods, which have been used in many research and development efforts [9,10,11].

There is also another type of method, which identifies fires using a daily change model (DTC) that estimates non-fire temperatures [12,13,14,15]. This type of method uses a robust fitting algorithm to remove anomalous data and a Kalman filter (KF) to build a highly accurate DTC model. A further approach is to use deep learning to combine geostationary meteorological satellite data with high-temporal and low-spatial resolution and polar-orbital satellite data with low-temporal and high-spatial resolution, in order to simultaneously take advantage of both types of data for fire progression monitoring [16]. A method to obtain parameters which are effective for fire detection has also been developed, by inputting a combination of differences and ratios of various Himawari-8 bands into a random forest classifier and selecting features of high importance [17]. Machine learning has attracted particular attention in recent years, and many studies have incorporated machine learning techniques into fire detection approaches [18,19,20]. Algorithms aimed at early fire detection by taking advantage of the high temporal resolution of geostationary satellites have also been studied [21,22,23,24]. Even when early detection is the goal, fire detection is still performed by estimating the temperature of the background region and separating fire pixels using a spatial or temporal approach. However, fires in the early stage are small in size and temperature, and the use of threshold judgements often lead to detection omissions. Table 1 lists existing fire detection methods using geostationary satellites.

Against this background, we propose an early fire detection method that extends the MOD14 algorithm. Early detection of forest fires and rapid extinguishing of fires will help to reduce the spread of damage caused by fires. For example, in Japan, the early detection of forest fires is conducted not by satellite remote sensing but by fire watchtowers and helicopter observations. In the case of helicopters, periodic patrols are conducted; however, in most cases, after a fire is detected by a local caller, a decision is made as to whether the fire is large enough to require a helicopter before a dispatch is requested, which requires human resources to make such a decision [25]. If satellite remote sensing can detect the onset and scale of a forest fire at an early stage, it is possible to efficiently determine whether or not a helicopter can be dispatched. Pixels in the early stages of a fire have a small difference from the background area in terms of brightness temperature, making identification with conventional threshold processing difficult. In addition, geostationary satellite sensors are more suitable than polar-orbiting satellite sensors, as a high temporal resolution is required for early fire detection; however, the low spatial resolution makes identification by conventional threshold processing even more difficult. Therefore, in this paper, we (1) develop an early stage forest fire detection algorithm that combines context testing in the MOD14 algorithm and machine learning, and (2) validate its effectiveness using Himawari-8 data including forest fires in their early stages.

## 2. Materials and Methods

### 2.1. MOD14 Algorithm

In the MOD14 algorithm [4,5], fires are detected through the following steps.

First, land and water pixels are separated using the land/water mask in the MODIS Geolocation Field (MOD03) product, and pixels that satisfy the following conditions are determined to be cloud pixels:

(1) $$\left(\rho_{0.65}+\rho_{0.86}>1,2\right)\;\mathrm{or}\;\left(T_{12}<265K\right)\;\mathrm{or}\;\left(\rho_{0.65}+\rho_{0.86}>0.7\;\mathrm{and}\;T_{12}<285K\right)\;\;\mathrm{or}\;(\mathrm{water}\;\mathrm{pixel}\;\mathrm{and}\;\rho_{0.86}>0.25\;\mathrm{and}\;T_{12}<300K),$$

where *ρ*_0.65_ and *ρ*_0.86_ are the top of the atmosphere (TOA) reflectance in the 0.65 and 0.86 μm bands, respectively, and *T*_12_ is the TOA brightness temperature in the 12 μm band.

Next, potential fire pixels are determined by the following condition:

(2) $$T_{4}>\bar{T_{4}}+5K\;\mathrm{and}\;\Delta T>\bar{\Delta T}+5K,$$

where *T*_4_ is the TOA brightness temperature in the 4 μm band, *∆T* is *T*_4_ minus TOA brightness temperature in the 11 μm band, and $\bar{T_{4}}$ and $\bar{\Delta T}$ are the average of *T*_4_ and *∆T* of pixels in the MODIS image centered on the pixel of interest, which are within 301 pixels horizontally and 30 pixels vertically and whose 4 μm band is less than 360 K.

Pixels that satisfy Equation (2)—that is, potential fire pixels—are finally determined by applying the context test, which determines whether a pixel is a fire or not based on its relationship to surrounding pixels. In the context test, a 3 × 3 window centered on each potential fire pixel is used as the initial size and the window is expanded sequentially up to a size of 21 × 21 until the land pixels that are not background fire (*T*_4_ > 325 K and Δ*T* > 20 K) in the window are more than 25% and 8 pixels in the window. Then, the mean value and mean absolute deviation of ∆*T*, among other values, within the window are calculated, and each potential fire pixel is determined to be a fire or not based on the following conditions:

(3) $$T_{4}>360K,$$

(4) $$\Delta T>\bar{\Delta T}+3.5\delta_{\Delta T},$$

(5) $$\Delta T>\bar{\Delta T}+6K,$$

(6) $$T_{4}>\bar{T_{4}}+3\delta_{4},$$

(7) $$T_{11}>\bar{T_{11}}+\delta_{11}-4K,$$

(8) $$\delta \prime_{4}>5K,$$

where *δ* is the mean absolute deviation of each variable. If a pixel satisfies Equation (3), or (4) through (7), or (4) through (6) and (8), it is determined to be a fire pixel.

### 2.2. Himawari-8 AHI Instrument

The geostationary meteorological satellite Himawari-8 was developed by the Japan Meteorological Agency (JMA), and launched by the Japan Aerospace Exploration Agency (JAXA) in October 2014. The Advanced Himawari Imager (AHI) onboard the Himawari-8 satellite has 16 bands ranging from visible to infrared bands, with a resolution of 500 m to 2 km, and it observes the Earth’s disk every 10 min and the Japanese region every 2.5 min. Although the AHI instrument has low spatial resolution, in comparison with other polar orbital satellite sensors such as the Moderate Resolution Imaging Spectroradiometer (MODIS) onboard the Terra and Aqua satellites and the Visible/Infrared Imager and Radiometer Suite (VIIRS) onboard the Suomi-NPP and JPSS1 satellites, it has high temporal resolution, which provides an advantage for fire detection in the early stage. Therefore, we developed an early fire detection method using images observed by the AHI instrument, although other geostationary satellite sensors can also be used, if they have similar specifications to the AHI instrument. Table 2 shows the basic specifications of the AHI bands used in this study. 

In this study, we used Himawari-8 AHI images which have been geometrically corrected and calibrated, downloaded from JAXA’s P-Tree site [26].

### 2.3. Proposed Method

#### 2.3.1. Overview

In this study, we propose a method for early fire detection from Himawari-8 AHI images with high temporal resolution using a modified MOD14 algorithm.

Pixels in the early stages of a fire have a small difference in brightness temperature from the background region and, so, it is difficult to distinguish them using conventional thresholding methods. Therefore, the proposed method entrusts the decision of threshold judgment in the context test of the MOD14 algorithm to a random forest classifier, such that fires that may be missed due to a slight difference in threshold judgment can be expected to be identified. In addition, weather information and band values are added to the input data for the random forest classifier, in order to assist in fire identification.

**Figure 1:**
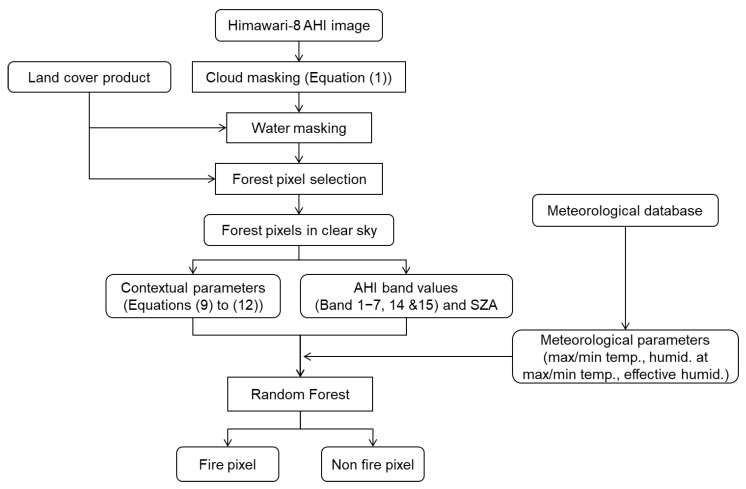
Processing flow of proposed method.

Figure 1 shows the processing flow of the proposed method. Cloud masking is performed using Equation (1) in the MOD14 algorithm. Water masking and forest pixel selection are performed using relevant land-cover products.

#### 2.3.2. Modification of MOD14 Algorithm

The MOD14 algorithm uses MODIS images and aims to detect all types of thermal anomalies, including forest fires. On the other hand, the proposed method uses Himawari-8 AHI imagery and aims to detect the early stages of forest fires. Thus, there are differences in sensor (i.e., band and spatial resolution) and observation target between the two. Therefore, we modified the MOD14 algorithm to enable early detection of forest fires using AHI imagery. The main modification is that, instead of determining the final fire pixel based on the results of the context test for potential fire pixels, as in the MOD14 algorithm, the parameters—including those related to the context test—are used as input into a random forest classifier, which makes the fire decision. Details on the modifications are given in the following.

(1) Exclusion of determining potential fire pixels

The proposed method aims to detect a fire pixel when its pixel value is still low, as it is associated with a fire that has just started. Therefore, the selection of potential fire pixels in the normal MOD14 algorithm can miss pixels with low pixel values. Therefore, the proposed method excludes the process of determining potential fire pixels.

(2) Modifications in background pixel search for context test

As the spatial resolution of the AHI instrument is lower than that of the MODIS instrument, it is difficult to obtain spatial contrast, as the increase in pixel value due to high temperature areas of the same area on the ground should be small. Therefore, to obtain more contrast with background pixels, a 3 × 3 pixel area centered on the pixel of interest is excluded from the effective pixels when calculating the average value of background pixels in the MOD14 algorithm. The exclusion of the pixels surrounding the pixel of interest is due to the effects of the Modulation Transfer Function (MTF) of the sensor. In addition, one of the search termination conditions for background pixels in the MOD14 algorithm, “25% or more of the window,” was also excluded.

The detection sensitivity of background fire pixels, which are excluded during the search for background pixels, will be also reduced due to the low spatial resolution of the AHI instrument. Therefore, if the condition “*T*_4_ > 325 K and Δ*T* > 20 K”, which is regarded as a background fire pixel in the context test of the MOD14 algorithm, is adopted as-is, background fire pixels may be missed. Therefore, this condition was changed to “*T*_4_ > 315 K and Δ*T* > 10 K,” in order to make it easier to detect background fire pixels.

(3) Context test for forest pixels only

The proposed method aims to enhance the early detection of forest fires. Therefore, in order to improve the detection accuracy, we modified the context test, limiting the pixels to be compared to only forest pixels, classified according to a land-cover product. Note that, when the difference in observed brightness temperature is evaluated between pixels with the same forest class, it is generally smaller than the difference between pixels with different classes. Therefore, in the proposed method, the threshold value “Δ*T* > $\bar{\Delta T}$ + 6 K” in the context test of the MOD14 algorithm was changed to “Δ*T* > $\bar{\Delta T}$ + 5.5 K.” This change makes it easier to detect small temperature changes caused by fires that occur in the forest.

(4) Fire decision by random forest classification

In the context test of the MOD14 algorithm, fire is determined through thresholding, by comparing the brightness temperature between the pixel of interest and the surrounding pixels. However, the proposed method introduces a random forest classifier to this fire determination process for more effective detection of early fires. First, for each of the four threshold determination Equations (4)–(7), we introduce new variables, defined by the difference between the two sides, as shown in each of the following equations:

(9) $$x_{1}=\Delta T-(\bar{\Delta T}+3.5\delta_{\Delta T}),$$

(10) $$x_{2}=\Delta T-(\bar{\Delta T}+5.5K),$$

(11) $$x_{3}=T_{4}-(\bar{T_{4}}+3\delta_{4}),$$

(12) $$x_{4}=T_{11}-\left(\bar{T_{11}}+\delta_{11}-4K\right).$$

We call these context parameters. For the random forest classifier, these context parameters; the observed values (TOA reflectance or TOA brightness temperature) of AHI bands 1–7, 14, and 15; the solar zenith angle (SZA); and five meteorological parameters—maximum air temperature (AT), minimum AT, relative humidity (RH) at maximum AT, RH at minimum AT, and effective humidity on that day—are used as input parameters for the random forest classifier. The effective humidity is an indicator of flammability of wood and other materials, and it was calculated using the following equation [27]:

(13) $$H_{e}=\left(1-r\right)(H_{0}+rH_{1}+r^{2}H_{2}+\ldots +r^{7}H_{7}),$$

where *H_n_* is the average humidity of the previous *n* days and *r* is a constant that represents the effects of past humidities. In this study, the average humidity was calculated by averaging the humidity at the maximum and minimum temperatures, *r* was given as 0.7, and *n* as 7.

### 2.4. Validation Study

#### 2.4.1. Study Area and Time Period

**Figure 2:**
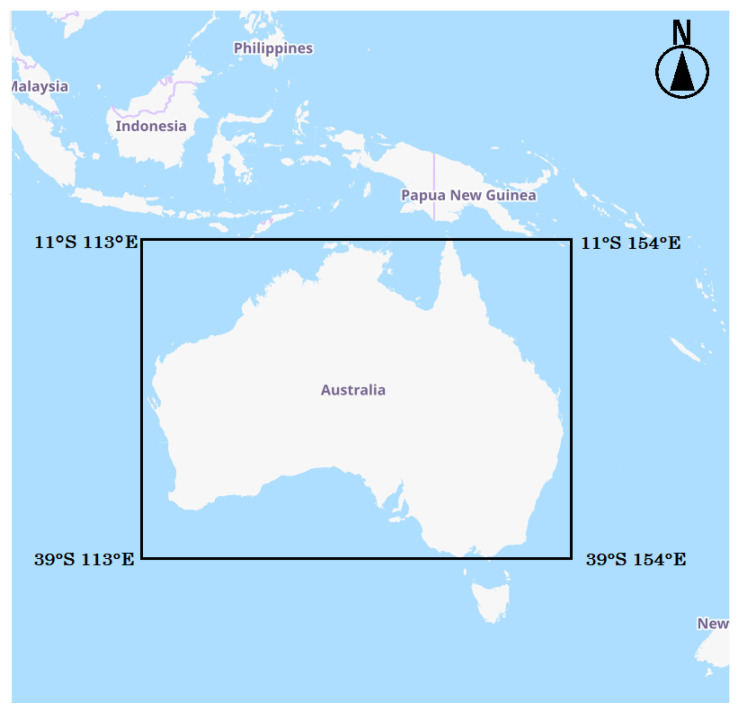
Location of study area (provided by OpenStreetMap).

Australia is a region where forest fires are likely to occur and grow in scale, due to low precipitation and abundant fire fuel trees. For example, in 2019–2020, a large forest fire in southeastern New South Wales caused significant damage [28]. Due to the need to collect a large number of AHI images observed immediately after a fire, the study area was defined as the entire Australian region, and the time period covered was from 2016 to 2021, including the time of the major fire event. Figure 2 shows the location of the study area.

#### 2.4.2. Meteorological Data

The meteorological data (max and min temperatures, and humidities at max and min temperatures) were obtained from a daily grid data set with a resolution of 5 km in the scientific information for land owners (SILO) database, which is a database of Australian climate data hosted by the Queensland Department of Environment and Science (DES) [29]. The effective humidity for each day was calculated from these values, using Equation (13).

#### 2.4.3. Forest Pixel Selection and Water Masking

Whether a pixel was forested or not was determined based on the land-cover classes provided in the Dynamic Land-Cover Data set (DLCD) Version 2 [30]. This database provides a total of 22 classes, including urban, shrub, and wetland, at a resolution of 250 m. In this study, pixels with class numbers 31 to 34 were selected as forests, after downscaling the resolution to match the 2 km spatial resolution of AHI images.

In addition, the MOD14 algorithm first identifies water bodies using the water mask of the MODIS Geolocation Fields (MOD03) product; however, in this study, the DLCD product was also used for the identification of water bodies. After downscaling to a spatial resolution of 2 km, those with class values of 0, 3, and 4 were selected as water bodies.

#### 2.4.4. AHI Images Used and Labeling

For training and validation of the proposed method, a large number of AHI images, including forest fires in their early stages, must be prepared and further labeled to distinguish between fires and non-fires.

First, we collected many MOD14 products over the study area in the period 2016–2021 from the Level-1 and Atmosphere Archive & Distribution System Distributed Active Archive Center (LAADS DAAC) [31]. Then, using the DLCD products, thermal anomaly pixels that had forest classes and were isolated were selected from each MOD14 product and recorded. For each recorded thermal anomaly pixel, the AHI images of bands 1–7, 14, 15 and SZA observed at the time closest to the thermal anomaly time were obtained, and sub-images of 256 × 256 pixels centered at the location of the thermal anomaly pixel were extracted from them. Here, the thermal anomaly pixel in each sub-image is not necessarily early fire; an early fire may have occurred before the time when it was detected as a MOD14 product. Therefore, the AHI images were collected and labeled according to the following procedure.

1. Time-series AHI images were acquired at 1 h intervals from the time of the thermal anomaly toward the past. Then, the modified MOD14 algorithm was applied to tentatively determine the presence or absence of a fire, and the time *t*_0_ was obtained when the fire was apparently no longer detected. The fire was assumed to have occurred within tens of minutes after time *t*_0_.
2. Time-series AHI images were acquired at 10 min intervals from time *t*_0_ to 1 h later. The time of fire occurrence was then tentatively estimated by thresholding based on the following equations:
  (14) $$T_{4}>\bar{T_{4}}+5K\;\mathrm{and}\;\Delta T>\bar{\Delta T}+5K,$$
  (15) $$T_{d}>\bar{T_{d}}+0.3K\;\mathrm{and}\;T_{d}>\bar{T_{d}}+4.0\delta_{d},$$
3. where *T_d_* is the brightness temperature change over 10 min in Band 7, and $\bar{T_{d}}$ and *δ_d_* are the mean value and mean absolute deviation within 7 × 7 pixels centered on the pixel of interest, respectively. A fire was considered to have occurred when either Equation (14) or (15) was satisfied, and the time when the fire was first detected was considered to be the tentative fire occurrence time.
4. To more accurately label the early stage fire, the spatial pattern changes in the AHI image were visually checked before and after the tentatively determined fire occurrence time, and the labels were modified as necessary to obtain the final label.
5. After labeling was completed for each fire, time-series AHI images at 10 min intervals for several 10 min periods before and after the time of the fire were used as the AHI image set for that fire, which were used for the evaluation described in Section 2.4.5.

#### 2.4.5. Performance Evaluation

For training and inference by the proposed method using the AHI image set for each fire, fire-labeled pixels were input into the modified MOD14 algorithm as fire pixels. In addition, a number of non-fire labeled pixels were randomly selected from each AHI image used and input into the algorithm as non-fire pixels. As a result, 1564 fire pixels and 2715 non-fire pixels were extracted from 3339 AHI images. In order to separate these for training and testing, the data from 2016–2019 were defined as the training set and the data from 2020–2021 as the testing set. As a result, the training data consisted of a total of 1208 fire pixels and 2074 non-fire pixels in 2576 AHI images, while the test data consisted of a total of 354 fire pixels and 641 non-fire pixels in 763 AHI images. The number of trees and number of layers were set to 100 and 20, respectively, as hyperparameters of the random forest for training.

The performance of the proposed method was evaluated according to its Accuracy, Precision, Recall, and F-measure, defined by the following equations:

(16) $$\mathrm{Accuracy}=\frac{TP+TN}{TP+FP+TN+FN}\times 100,$$

(17) $$\mathrm{Precision}=\frac{TP}{TP+FP}\times 100,$$

(18) $$\mathrm{Recall}=\frac{TP}{TP+FN}\times 100,$$

(19) $$F-\mathrm{measure}=\frac{2TP}{2TP+FN+FP}\times 100,$$

where *TP* denotes the pixels correctly determined as fire, *TN* denotes the pixels correctly determined as non-fire, *FP* denotes the pixels incorrectly determined as fire, and *FN* denotes the pixels incorrectly determined as non-fire.

To validate the proposed method using these data, we applied the proposed method in three ways: (A) all parameters (nine AHI band values, SZA, four contextual parameters, and five meteorological parameters) were used as input, (B) only AHI band values and SZA were used as input, and (C) only four contextual parameters were used as input.

For further comparison with existing methods, the above data set was also applied to the method proposed by Jang et al. [17]. This method selects features of high importance in fire detection, using a random forest classifier, from among the various differences and ratios of multiple Himawari-8 bands. In this study, we used the 26 features, shown in Table 3, selected for the Korean test site [17]. As the objective of this study is to detect early stage forest fire pixels, the determination of potential fire pixels by thresholding in their method was excluded in our evaluation study, due to the high possibility of missing low-temperature pixels found in the early stage fire.

## 3. Results

The evaluation results for each method are shown in Table 4. While the method of Jang et al. method achieved 67.83% precision, 42.37% recall, and 52.17% F-measure, the proposed method using all parameters (Case A) had 86.09% precision, 92.66% recall, and 89.25% F-measure, showing good detection accuracy. In particular, the recall exceeded 90%, indicating that the proposed method was able to detect many fire pixels in the early stages of the fire, while the precision was below 90%, resulting in false fire detection. The accuracy of the proposed method using only context parameters (Case C) was slightly lower than that of Case A, while the accuracy of the proposed method using only band values (Case B) was significantly lower, indicating that the context parameters have a significant impact on improving accuracy.

**Figure 3:**
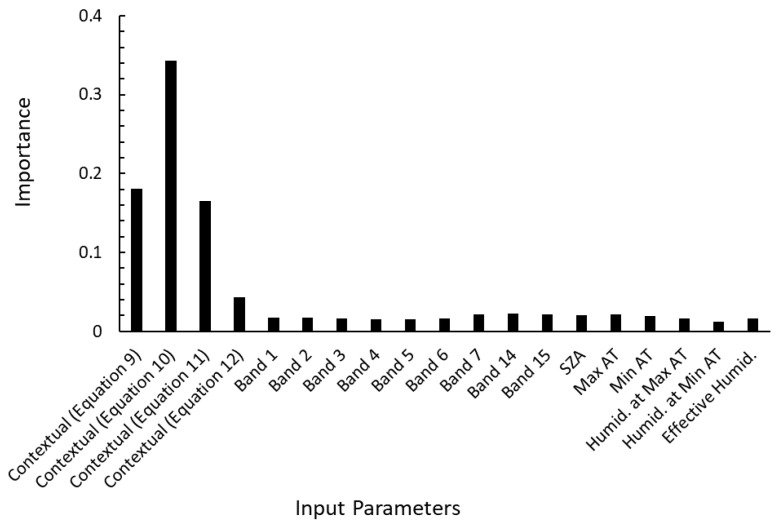
Importance of each parameter given to the random forest classifier in the proposed method.

We calculated the importance of each parameter given to the random forest classifier for the proposed method using all parameters, as shown in Figure 3. The four contextual parameters, especially Equation (10), followed by (9) and (11), were found to have significant impacts on the random forest classification. This result is consistent with the higher accuracy of the methods using contextual parameters, detailed in Table 4.

**Figure 4:**
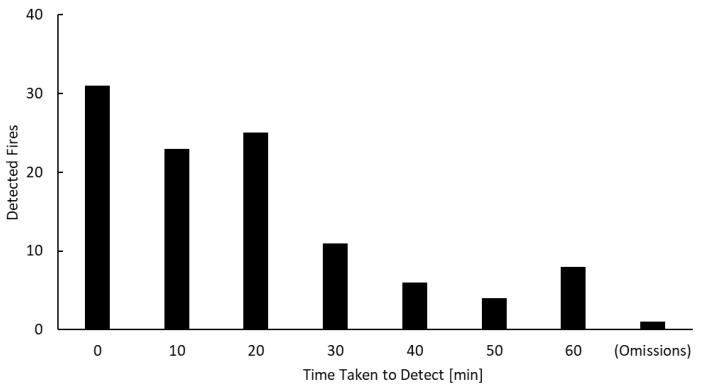
Histogram of the time taken by the proposed method using all parameters to detect a fire after it has occurred, at 10 min intervals.

Figure 4 shows the time taken by the proposed method using all parameters to detect a fire after it had occurred, at 10 min intervals. The proposed method was able to detect 108 out of 109 fires within 60 min of their occurrence. The average time from the time when the fire was estimated to have occurred to the time when the fire could be detected by the proposed method was 18 min. These results indicate that the proposed method successfully detected the early stages of a fire. On the other hand, for fires with slow time variation of temperature, detection was delayed, as it takes more time for the fire pixel to rise to a temperature at which it can be separated from the background area.

**Figure 5:**
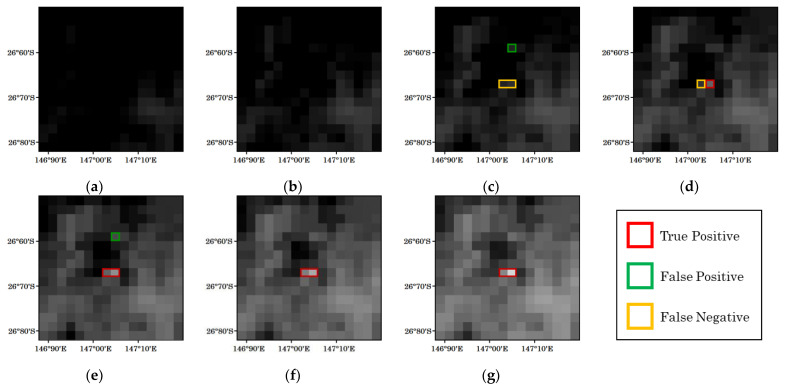
Example of detection results for a forest fire that occurred at 0:10 UTC on 27 May 2021. The observation times for each figure are (**a**) 23:50 (previous day), (**b**) 00:00, (**c**) 00:10, (**d**) 00:20, (**e**) 00:30, (**f**) 00:40, and (**g**) 00:50.

Although the detection was basically successful, there were cases where some problems were observed. As an example, Figure 5 shows the detection results of the proposed method for a forest fire that occurred at 0:10 UTC on 27 May 2021. The detection was able to be performed 10 min after the onset of the fire. It seems that the pixel temperature did not rise sufficiently at 0:10 (the time of the fire), such that the temperature difference from the background forest pixels was too small to detect the fire. False fire detection was also observed at 0:10 and 0:30. Although these were not fires, the temperature was higher than that of the background area, so they were falsely detected as fires.

**Figure 6:**
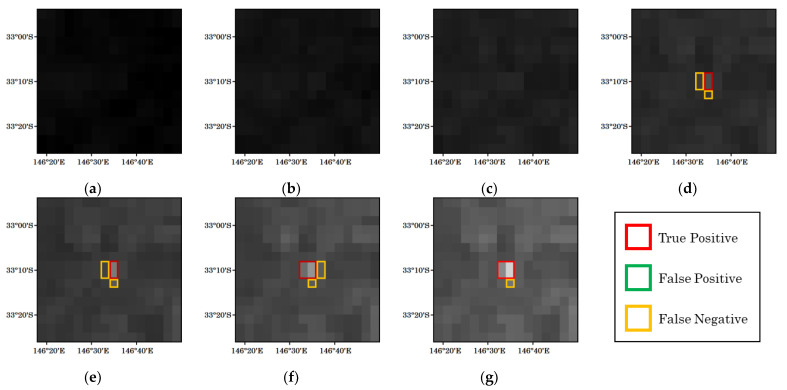
Example of detection results for a forest fire that occurred at 21:00 UTC on 18 February 2021. The observation times for each figure are (**a**) 20:30, (**b**) 20:40, (**c**) 20:50, (**d**) 21:00, (**e**) 21:10, (**f**) 21:20, and (**g**) 21:30.

Figure 6 is another example, showing the detection results of the proposed method for a forest fire that occurred at 21:00 UTC on 18 February 2021. In this example, the detection was successful immediately after the fire occurred. However, there were detection omissions around the pixels that were determined to be fire. These pixels are fires located far from the center of the fire, and the observed brightness temperature was lower than that in the center of the fire.

## 4. Discussion

Early stage fires are characterized by the fact that the pixel values of fire pixels are not high, as the fire area is small and the pixel value difference between the fire pixels and the surrounding background pixels is low, which are especially noticeable in low-spatial-resolution data such as Himawari-8 AHI imagery. As the purpose of this study was to detect early stage fires, a data set containing many early stage fires was created using a time-reversal approach based on MOD14 products. Fire detection evaluation based on such a data set has not been carried out in many previous studies. In fact, the method of Jang et al. [17] has been shown to provide highly accurate evaluation results in fire detection using Himawari-8 data; however, the 26 pixel-based features they proposed did not provide high accuracy in this early stage fire data set. The same was true when only band values were given to the random forest classifier in the proposed method, which showed even lower accuracy results than the method of Jang et al., due to the smaller number of features.

On the other hand, when the contextual judgments of the MOD14 algorithm were converted into features as contextual parameters and given to the random forest classifier, the accuracy was remarkably improved on the same data set. This indicates that contextual judgment based on comparison with surrounding pixels is superior to pixel-based judgment for early stage fire detection. This contextual judgment is similar to the process by which humans visually detect a fire from a slight temperature increase. In this study, to further improve accuracy over the method of inputting only contextual parameters into the random forest classifier, we also incorporated pixel-based features (each band value) and meteorological features (i.e., maximum/minimum temperature, humidity at maximum/minimum temperature, and effective humidity) as input parameters for the random forest classifier. The incorporation of these parameters into the random forest classifier improved the accuracy of detection. However, the improvement in accuracy was small (less than 1%), as indicated by the high importance of the four context parameters (especially Equation (10), followed by (9) and (11)) in the importance analysis of the parameters in the random forest classifier.

The obtained results indicate that the proposed method using a random forest classifier with contextual parameters is effective for early stage forest fire detection and that the detection accuracy can be slightly increased by providing additional band values and meteorological data as features.

## 5. Conclusions

In this study, we proposed a method for detecting early stage forest fires from low-spatial-resolution AHI images. The proposed method parameterizes the four contextual conditions of the MOD14 algorithm as features and detects early stage fires from forest areas by inputting these parameters, nine AHI band values, SZA, and five meteorological values into a random forest classifier. To evaluate the proposed method, we created an early stage forest fire data set in Australia using a time-reversal approach with MOD14 products and time-series AHI images, and we applied the proposed method and the method of Jang et al. to this data set for comparison. The results indicated that the proposed method excluding contextual parameters and the method of Jang et al. [17] did not achieve sufficient accuracy on this data set, while the proposed method including contextual parameters detected forest fire pixels in the early stage with about 90% precision and recall. In addition, the importance analysis of the constructed random forest classifier demonstrated that the contribution of contextual parameters was particularly large.

The proposed method can be applied to geostationary satellite sensors other than the Himawari-8 AHI instrument, as well as polar-orbiting satellite sensors. We expect that this method will be used as an effective method for forest fire detection.

## Figures and Tables

**Table 1:** Existing methods using geostationary satellites.

Authors	Satellite-Sensor	Method
Koltunov, A. et al. (2016) [22]	GOES-ABI	Multi-temporal and contextual test
Carolina, F. et al. (2017) [23]	MSG-SEVIRI	Multi-temporal change-detection
Biase, V.D. et al. (2018) [24]	MSG-SEVIRI	Contextual analysis and change-detection
Jang, E. et al. (2019) [17]	Himawari-8-AHI	Threshold test and random forest classification
Xie, Z. et al. (2018) [14]	Himawari-8-AHI	Temporal and spatial analysis
Wickramasinghe, C. et al. (2018) [9]	Himawari-8-AHI	Contextual analysis and change-detection
Hong, Z. et al. (2022) [20]	Himawari-8-AHI	Novel convolutional neural network
Xu, G. et al. (2017) [7]	Himawari-8-AHI	Contextual analysis

**Table 2:** Basic specifications of AHI bands used in this study.

Band	Central Wavelength (µm)	Spatial Resolution (km)
1	0.471	1
2	0.510	1
3	0.639	0.5
4	0.857	1
5	1.61	2
6	2.26	2
7	3.89	2
14	11.2	2
15	12.4	2

**Table 3:** The 26 features suggested by Jang et al. [17]. Chx is the TOA radiance of AHI band x, and BTx is the TOA brightness temperature of AHI band x.

Type	Feature
TOA radiance based	Ch5–Ch7, Ch6–Ch7, Ch4–Ch7, Ch7, Ch7–Ch15, Ch12–Ch15, Ch7–Ch12
TOA brightness temperature based	BT13/BT15, BT7/BT13, BT7–BT13, BT7–BT14, BT13–BT15, BT7/BT14, BT7–BT11, BT7/BT11, BT7–BT15, BT7–BT12, BT7/BT12, BT12–BT16, BT7, BT7–BT14, BT7/BT15, BT7/BT16, BT7/BT10, BT7/BT9, BT9/BT16

**Table 4:** Results of performance evaluation study for the proposed method (three cases) and the existing method (i.e., the method of Jang et al. [17]).

Method	Accuracy	Precision	Recall	F-Measure
Proposed method with all parameters (Case A)	92.06%	86.09%	92.66%	89.25%
Proposed method with only AHI band values and SZA (Case B)	65.53%	52.79%	29.38%	37.75%
Proposed method with only context parameters (Case C)	91.36%	85.26%	91.53%	88.28%
Existing method (Jang et al. [17])	72.36%	67.83%	42.37%	52.17%

## Data Availability

Not applicable.

## Abbreviations

The table below shows the main symbols used in this study.

$\rho_{0.65}$	TOA reflectance in the 0.65 μm band
$\rho_{0.86}$	TOA reflectance in the 0.86 μm band
$T_{4}$	TOA brightness temperature in the 4 μm band
$T_{11}$	TOA brightness temperature in the 11 μm band
$T_{12}$	TOA brightness temperature in the 12 μm band
ΔT	$\mathrm{Brightness}\;\mathrm{temperature}\;\mathrm{difference}\;(=T_{4}-T_{11})$
$T_{d}$	Brightness temperature change over 10 min in the 4 μm band
$\bar{T_{4}}$	Mean brightness temperature in the 4 μm band
$\bar{T_{11}}$	Mean brightness temperature in the 11 μm band
$\bar{\Delta T}$	Mean brightness temperature difference
$\bar{T_{d}}$	Mean brightness temperature change over 10 min in the 4 μm band
$\delta_{4}$	MAD brightness temperature in the 4 μm band
$\delta_{11}$	MAD brightness temperature in the 11 μm band
$\delta_{\Delta T}$	MAD brightness temperature difference
$\delta_{d}$	MAD brightness temperature change over 10 min in the 4 μm band
$\delta \prime_{4}$	MAD brightness temperature in the 4 μm band rejected as background fires
$H_{e}$	Effective humidity
TOA	top of the atmosphere
MAD	mean absolute deviation
